# DNA as in‐formation

**DOI:** 10.1002/wfs2.1470

**Published:** 2022-08-15

**Authors:** Mareile Kaufmann

**Affiliations:** ^1^ Department of Criminology and Sociology of Law University of Oslo Oslo Norway

**Keywords:** DNA, Digital forensics, information, technology

## Abstract

Traces are fundamental vectors of information. This is the first of seven forensic principles formulated by the 2022 Sydney declaration. To better understand the *trace as information*, this article proposes the notion of *in‐formation*. DNA is matter in becoming. DNA changes as it travels across forensic sites and domains. New formations occur as humans, technologies and DNA interact. Understanding DNA as in‐formation is of particular relevance vis‐à‐vis the increase of algorithmic technologies in the forensic sciences and the rendering of DNA into (big) data. The concept can help identifying, acknowledging and communicating those moments of techno‐scientific interaction that require discretion and methodical decisions. It can assist in tracing what form DNA will take and what consequences this may have.

This article is categorized under:

Crime Scene Investigation > From Traces to Intelligence and Evidence
Forensic Biology > Ethical and Social Implications
Forensic Biology > Forensic DNA Technologies

## Graphical abstract

Digital forensic technologies change the ways in which DNA is processed as information. Understanding DNA as in‐formation can help us to assess the consequences of this shift. “View of the cell nucleus” by istockphoto.

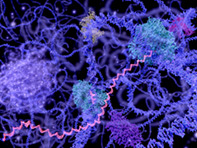

## 1 INTRODUCTION

In 2022, the Sydney declaration proposed a set of seven principles to define the essence of forensic science. Already the first principle suggests that the central forensic object of the *trace* is *information*: “Activity and presence produce traces that are fundamental vectors of information” (Roux et al. 2022, p. 332). Traces are “capable of being detected, recovered, examined and interpreted” (ibid.). This statement bears a sense of urgency to better understand the *trace as information*. I propose the notion of in‐formation to further qualify the vectorial character of the trace. This is important, because the very way in which traces are in‐formation has consequences for forensic results, practice and politics. Forensic understandings of traces and the tools that help extracting and analyzing them are not neutral entities, but always imply appraisals, decisions and adjustments that channel the vector of information and its eventual meaning. Consequences vary from juridical effects such as incrimination and exculpation (McCartney 2006) to more abstract problems such as the production of meaning from biological data (Stevens 2016). When we reflect how traces are in‐formation we can identify the points at which important decisions are taken and co‐produced by humans and machines. These are decisions that need to be communicated across professional boundaries to enable an assessment of their potential implications. In order to develop this argument, I revert to an idiosyncratic trace of the forensic sciences: DNA.

How we understand DNA as information is not only dynamic, but the status of DNA as information is also subject to dispute. Early DNA controversies, for example, involved considerable disagreements about the procedures for DNA sequencing (Lynch et al. 2008). Different techniques would yield different results. One of the key criticisms was that DNA matches are probabilistic, not absolute information (Cole and Lynch 2010). Scholars discussed whether and how DNA was considered meaningful enough to be invoked in courtrooms. These debates went on until an “inversion of credibility” came about, where the meticulous vetting processes of DNA traces as well as probabilistic error rates emerged as a strength (ibid.: 107). This inversion, then, was in part a result of the increasing integration of DNA with computational technologies. DNA was in‐formation.

While DNA analysis has always been dependent on digital technologies for extraction and visualization (Lynch et al. 2008, pp. 23–38), we now witness a change in the dimension and scale of technology use. Ever‐more DNA is collected and stored, and analyzing big genomic data (Murphy 2018) with algorithms is becoming a new normal. New technologies are developed for DNA sequencing at the crime site. We find software for DNA phenotyping to make identikit pictures. The rising number of DNA databases constantly provide new data to be analyzed. The increasing confluence of biology and technology has enormous potential for innovation, but also confronts researchers with new challenges. The use of new digital means adds a layer of complexity to biological projects and spending time on analyzing this extra layer is costly (Leonelli 2016).

Not only scientists formulate issues related to the ways in which DNA is in‐formation. In 2018, Norway's Federal Prosecutor General declared that there is a need for in‐depth research about DNA processing and other digital technologies in legal genetics (Petersen and Borg Ole 2018). In a 2019 report the UK Science and Technology Select Committee traced how the forensic sciences are in a state of crisis, finding that, amongst other things, the interpretation of the meaning of DNA across professions is in need of more streamlining (2019, p. 32).

Grasping these changing links between biology and technology requires more than commentary and critique. As Nicholas Rose suggests (2013), efforts are needed to decipher the operative philosophies of such processes. A key to deciphering these philosophies and their consequences brings us back to the concept of information. How does the increasing integration of DNA with digital technologies change DNA as information? What does that mean for forensic processes today? In suggesting an understanding of DNA as in‐formation, the article provides perspectives that can inspire reflection about the vector of DNA in digitized environments.

## 2 DNA IN FORMATION

The co‐emergence of molecular biology and information science in the 1960s was an important foundational development for the conception of DNA as information. Lily Kay argues that it was in the wake of these changes that “biological specificity became informational, and information, message and code eventually became biological concepts” (Kay 2000, p. 22). Today, DNA is often narrated as “central information storage system” (National Human Genome Research Institute, 2022) and as carrier of genetic information and instructions (Zhang et al. 2019). Even though we are very familiar with this image of DNA as information, there are different positions about the actual character of that information. In theoretical biology we find the stance that DNA is the “arbitrary abstraction of a material property” (Wills 2016a, p. 20150417). This understanding parallels theories that consider information as essentially semantic, as immaterial universal code (Floridi 2010). If information is conceptualized as an abstracted essence, it does suggest itself to think of DNA as typefied code. Such a code would render objects and processes computable (Cartwright et al. 2016) or even clonable (Floridi 2010). Indeed, this idea of the “pure information object, unfettered by matter” (Paul 2009, p. 19) nurtures the understanding of DNA as a driver of “functional information processing within cells, that is, computation” (Wills 2016a, p. 20150417; Neumann 1949). This view does, however, present the natural sciences with a conundrum: how can DNA as information—despite its abstract nature—serve as cause of biological events in the physical world? At the same time do theoretical biologists suggest that DNA sequence information “fuses” with the molecular componentry that generates meaning from information (Wills 2016b). That is to say, in what ways DNA is after all part of the molecular structure of cells—its material building blocks—and how exactly DNA is translated into phenotypes cannot be grasped with theories that consider information as abstracted essence. Perspectives from the field of new materialism adopt an alternative view: any information “must always be instantiated in a medium” to exist as information (Hayles 1999, p. 13; cf. Bennett 2010, Bowker et al. 2010, Blanchette 2011, Drucker 2013, Lupton 2016, Kaufmann and Jeandezboz 2017, Camus and Vinck 2019; Ribes 2019). Genetic information, then, cannot be without the materiality of DNA. It is literally embodied. Not just that, but DNA is matter in becoming: new formations occur when humans, technologies and DNA interact (Haraway 2015).

The very sequences of As, Cs, Ts, and Gs can be changed with engineering efforts, but also the ways in which epigenetics influence the interplay of DNA sequences and molecular componentry results in different physical formations. DNA is in‐formation when it is purified, filtered, dissolved in an enzyme, amplified or dyed for electrophoresis—the process of determining patterns of As, Cs, Ts, and Gs. Now that DNA is increasingly integrated with new cultures of measuring, it is in‐formation in new ways. Already the fact that DNA is captured by computers is a material re‐instantiation. Sequences materialize as ones and zeros in databases that can be searched by algorithms. DNA is in‐formation when it is subjected to large‐scale correlative analyses that assign meaning, for example physiognomic expressions, to specific sequences and DNA becomes an indicator for phenotypes. The normalization of DNA as digital information is arguably a substantial influence factor regarding *vectors of information*. I will illustrate these shifts briefly in relation to three technological phenomena: hardware, databases, and software.

## 3 THE IMPACT OF DIGITAL TECHNOLOGIES ON DNA

### 3.1 Hardware

How forensic procedures emerge as a standard is not always a straightforward process and choices taken here influence the outcome of analyses (Lynch et al. 2008). Already basic routines for collecting DNA at the crime site determine how information comes to matter in criminal cases. These routines are tightly linked to the choice of sequencing hardware and technique, which are a recurring subject of debate in the forensic sciences. Hardware to extract, sequence, quality control and visualize DNA are getting smaller in size. *Nanopore's MinION*, for example, seeks to offer higher processing power, high performance, and high‐throughput sequencing directly at the site. This promise directly relates to the trace as in‐formation.

Since results differ across products, the choice and performance of sequencers impact DNA in‐formation. Forensic communities address these differences by testing hardware, for example, in relation to their performance on degraded DNA (Sharma et al. 2020). While some solutions work well on degraded DNA, others do not. The choice of sequencer is thus an important influence factor in the formation of the trace. Another promise of new sequencers is “the potential to identify a perpetrator within hours” (Mapes et al. 2019, p. 29). Such tools for rapid analysis, however, tend to be “less sensitive than traditional technologies used in forensic laboratories” (ibid.). This confronts forensic scientists with the decision to either provide preliminary results rapidly or higher‐quality results later. This choice, too, can be decisive for the vector of information. What emerges as an additional layer of influence is that these decisions tie in with commercial interests. There is a greater collaboration between public and private service providers, which redefines how scientists, corporations and technologies collaborate in the informationalization and formation of traces. Due to the increasing professionalization and commercialization, this collaboration becomes very complex. What becomes clear from these examples is that hardware and related methodical decisions stabilize vectors of information.

### 3.2 Databases

Once collected, DNA is stored. DNA stays in‐formation when it is re‐embodied in different databases. In the past years, we could witness a growth in the number of DNA databases. These include national solutions for law enforcement for which the FBI's Combined DNA Index System (CODIS) emerges as a central program of support. Besides the many DNA databases for scientific and criminalistic research, private databases are on the rise, too. Many of them are tied to ancestry research, such as *23andMe*, *MyHeritage*, or *AncestryDNA*. The rise of DNA databases not only normalizes the collection of DNA, but also increases data available for analysis. The amount of available data, again, influences which algorithmic solutions can be used on datasets and how. Hence, collaborations between different database and technology providers occur. *GEDMatch* genealogy solutions collaborate, for example, with *VEROGEN* systems for preparing, sequencing, and analyzing DNA. As a result of this trend, police forces, scientists, corporations, and lay people have more comprehensive access to DNA, but also more complex design setups.

The growth of DNA databases, their accessibility and their integration with work processes can be of great assistance to crime solving. These shifts in DNA collection and storage may even render it possible to crowdsource some aspects of crime solving (MIT Technology Review 2019), but it also broadens bio‐surveillance. Scholarly discussions mainly focus on the sensitivity of data (e.g., Wienroth et al. 2014; Chen 2018), which is one of the more prevalent criticisms of this trend. Another important aspect is less reflected about, namely that databases differ. Decision‐making processes structure what exact information is collected and stored, and how it is stored and categorized, as well as who will be allowed to work with it (Murphy and Tong 2020). These differences in database design fundamentally affect the ways in which DNA can be compared and analyzed. Databasing, too, is a part of how DNA is in‐formation. What is more, storage procedures contribute to DNA staying in continued formation. Digital data are notoriously difficult to destroy as they travel and re‐materialize in different databases. The same was found for DNA data (Skinner and Wienroth 2019). The same DNA data, then, can be subjected to different types of analysis, leading to ever‐new formations.

### 3.3 Software

The availability of DNA in digital databases also enables new analytic approaches. The past years provided us with insights into the dynamics of big data and algorithms within media and politics (e.g., Boyd & Crawford 2012; Amoore 2018; Friedman & Nissenbaum 1996). In the context of forensics, this move presents us with new analytic procedures that yield considerable potential for medical, forensic and bioinformatical use. While the main aim of early genomic analyses in the context of law enforcement was to establish DNA matches and differences, algorithms are now increasingly used to infer traits from the genome. The approach is thus not only to compare different DNA samples, but to predict, for example, typical physical features and age from a sample. This information can be of valuable assistance in investigations, at the same time as it poses new challenges: it moves the focus from individual suspects to suspicious groups (M'charek and Wade 2020), where the use of skin color and morphology can reinforce stereotypical conceptions of race (M'charek 2020, Hopman 2021). It also introduces procedures that open the door for new forms of profiling that will require thorough ethical assessment. New profiling could, for example, include inferences about neurochemical conditions that may be expressed as behavioral traits, a form of ‘‐typing’ that could become highly controversial in law enforcement contexts.

Phenoptyping is a process where DNA is subjected to algorithms to infer observable characteristics. Estimates about the pigmentation of eye, hair and skin color arise from comparisons of genes known to impact pigmentation with large data pools of individuals whose geno‐ and phenotype is known. Based on this input data, an algorithm creates correlations between pigmentation genes and physiognomic expressions. These readings are then organized into categorical classifications (Hopman 2021). A similar process is used to infer morphological traits. DNA is in‐formation as a  phenotype is inferred from a sequence. These analytic procedures, too, are embedded in an established scientific culture of testing hypotheses and addressing error in tools and procedures (Lippert et al. 2017; Goodwin 2015; for familial analysis see Pilli et al. 2022). This culture of recognizing and dealing with procedural uncertainties tends to be underestimated in the critical discussions on phenotyping. At the same time, the very fact that this type of analysis is a scientific advance also creates the popular image of phenotyping as “an exercise in purely objective, indisputable science” (Murphy 2008, p. 490), where DNA is a stable type of information. Erin Murphy reminds us, however, that “DNA typing—done perfectly and precisely according to protocol—still often entails making discretionary calls and choices” (Murphy 2008, p. 491). For example, decisions need to be taken about how to categorize expressions of pigmentation and how to standardize morphological features (Hopman 2021). The software needs to assign visual values to DNA samples despite uncertainty about what can be considered noise and what is actual signal in the sample. Bridging this discrepancy between a scientific culture of testing and decision‐making and its popular image is a difficult communicative task for the forensic sciences. Understanding DNA as in‐formation can assist in communicating the moments of discretion in the vector of information. What is more, once these moments are acknowledged they can inform discussions about issues related to accountability and scientific conduct (Granja and Machado 2020), the risk of biases (Hopman and M'charek 2020) or other ethical, legal, and social issues (Toom et al. 2016).

## 4 CONCLUSION

DNA does not speak for itself. It never did. Today, digital technologies are more than ever part of making DNA speak. DNA is collected and rendered into digital data by hardware. It is stored in a variety of databases and analyzed by algorithms. These techno‐scientific dynamics matter as they create important shifts within forensic procedures. Together, technologies, scientific procedure and human discretion co‐produce how DNA speaks as information and what meaning it will have.

Understanding the forensic object of the trace as in‐formation can help identify and assess decisive moments that influence forensic processes. The notion of in‐formation takes as its vantage point the Sydney declaration's principle of the trace as a key *vector of information* and further qualifies that vectorial, evolving character of information. The dynamics of being in‐formation embrace the established forensic culture of testing hypotheses and addressing errors as well as the scientific and political nature of decision‐making involved in these procedures.

This article serves as a reminder that DNA is also in‐formation as big data and algorithmic technologies become normalized in forensic sciences. These technologies, too, entail design decisions and moments of discretion that determine what form DNA will take and what consequences this will have. Clear communication about these moments is needed. DNA as in‐formation is here also an auxiliary to acknowledge those decisions, adjustments and moments of discretion that need communication across professions and vis‐à‐vis the public.

## AUTHOR CONTRIBUTIONS

**Mareile Kaufmann:** Conceptualization (lead); funding acquisition (lead); methodology (lead); writing – original draft (lead); writing – review and editing (lead).

## FUNDING INFORMATION

This project has received funding from the European Research Council (ERC) under the European Union's Horizon 2020 research and innovation program (Grant agreement No. 947681).

## RELATED WIREs ARTICLE

Privacy implications of the new "omic" technologies in law enforcement

## FURTHER READING

None (all suggestions for further reading are part of the references, but I can highlight the most important ones if desired)

## Data Availability

Data sharing is not applicable to this article as no new data were created or analyzed in this study.
